# Flexibility of the Linker between the Domains of DNA Methyltransferase SsoII Revealed by Small-Angle X-Ray Scattering: Implications for Transcription Regulation in SsoII Restriction–Modification System

**DOI:** 10.1371/journal.pone.0093453

**Published:** 2014-04-07

**Authors:** Petr V. Konarev, Galina S. Kachalova, Alexandra Yu Ryazanova, Elena A. Kubareva, Anna S. Karyagina, Hans D. Bartunik, Dmitri I. Svergun

**Affiliations:** 1 European Molecular Biology Laboratory, Hamburg Outstation, Hamburg, Germany; 2 Bach Institute of Biochemistry, Russian Academy of Sciences, Moscow, Russia; 3 Belozersky Institute of Physico-Chemical Biology, Lomonosov Moscow State University, Moscow, Russia; 4 Gamaleya Institute of Epidemiology and Microbiology, Moscow, Russia; 5 Institute of Agricultural Biotechnology, Russian Academy of Sciences, Moscow, Russia; Institute of Enzymology of the Hungarian Academy of Science, Hungary

## Abstract

(Cytosine-5)-DNA methyltransferase SsoII (M.SsoII) consists of a methyltransferase domain (residues 72–379) and an N-terminal region (residues 1–71) which regulates transcription in SsoII restriction–modification system. Small-angle X-ray scattering (SAXS) is employed here to study the low resolution structure of M.SsoII and its complex with DNA containing the methylation site. The shapes reconstructed *ab initio* from the SAXS data reveal two distinct protein domains of unequal size. The larger domain matches the crystallographic structure of a homologous DNA methyltransferase HhaI (M.HhaI), and the cleft in this domain is occupied by DNA in the model of the complex reconstructed from the SAXS data. This larger domain can thus be identified as the methyltransferase domain whereas the other domain represents the N-terminal region. Homology modeling of the M.SsoII structure is performed by using the model of M.HhaI for the methyltransferase domain and representing the N-terminal region either as a flexible chain of dummy residues or as a rigid structure of a homologous protein (phage 434 repressor) connected to the methyltransferase domain by a short flexible linker. Both models are compatible with the SAXS data and demonstrate high mobility of the N-terminal region. The linker flexibility might play an important role in the function of M.SsoII as a transcription factor.

## Introduction

DNA methyltransferases (MTases) catalyze methyl group transfer from *S*-adenosyl-*L*-methionine (AdoMet) to a certain base in DNA (cytosine or adenine). The cofactor AdoMet is converted into *S*-adenosyl-*L*-homocysteine (AdoHcy) in this reaction. Bacterial DNA MTases can be divided into the following 3 classes: (cytosine-5)-DNA MTases (C5-DNA MTases), N4-cytosine-DNA MTases, and N6-adenine-DNA MTases which methylate C5 atom of cytosine, N4 atom of cytosine, and N6 atom of adenine, respectively. Most known prokaryotic DNA MTases are components of Type II restriction–modification (R–M) systems which protect host cells from bacteriophage infection. A common Type II R–M system consists of a MTase which methylates certain DNA sequences and a restriction endonuclease (RE) which hydrolyses DNA if these sequences remain unmodified. An excessive RE activity can be dangerous for the host cell and the expression of the RE and MTase genes should thus be strictly coordinated [1]. To date, over 4000 R–M systems are characterized biochemically and/or genetically and over 20000 R–M systems are predicted bioinformatically (see REBASE) [2]. Among them, 3 variants of gene expression control at the transcriptional level are recognized: by a special C (controller) protein, by the MTase enzymatic activity, and by the MTase binding to a special regulatory site which differs from the methylation site [3].

The first method of gene expression control is based on the presence of a small gene encoding C-protein. This protein binds to an operator DNA sequence and regulates expression of its own gene as well as expression of the RE and MTase genes. Up to now, crystal structures of 4 different C-proteins are solved: C.AhdI [4], C.BclI [5], C.Csp231I [6], and C.Esp1396I [7]. All of them share highly similar three-dimensional structure including a classical helix–turn–helix (HTH) motif and are assigned to the Xre (xenobiotic response element) family of transcription regulators. All these C-proteins are dimeric in the crystallized form and C.AhdI has been shown to be a dimer in solution as well [8].

The second variant of gene expression control occurs for instance in the R–M system CfrBI. The MTase gene has a strong promoter which overlaps with a weak promoter of the RE gene. A single methylation site, in turn, overlaps with the −35 promoter element of the MTase gene. Thus, the MTase enzymatic activity leads to methylation of the −35 element which provides the MTase gene repression and stimulates transcription of the RE gene [9].

The SsoII R–M system from *Shigella sonnei* has the third variant of gene expression control. The MTase of this R–M system, M.SsoII, is the main object of the present study. It belongs to C5-DNA MTases and methylates the second cytosine nucleotide (underlined) in the sequence 5′-CCNGG-3′/3′-GGNCC-5′ [10], [11]. M.SsoII can also act as a transcription factor binding to a 15-bp quasipalindromic sequence 5′-AGGACAAATTGTCCT-3′/3′-TCCTGTTTAACAGGA-5′ (the regulatory site) in the intergenic region of the SsoII R–M system and therefore downregulating the expression of its own gene and stimulating the expression of the cognate RE gene [12], [13]. The same mechanism of action is shown for M.Ecl18kI [14], which differs from M.SsoII by a single amino acid residue. Some other C5-DNA MTases are shown experimentally to repress their own genes without any impact on expression of the corresponding REs, namely M.EcoRII [15], [16], M1.LlaJI [17], M.MspI [18], and M.ScrFIA [19].

Sequence analysis demonstrates that M.SsoII contains 2 domains: a typical C5-DNA MTase domain (residues 72–379) and a regulatory domain (RD, residues 1–55). The latter one is predicted to contain an HTH motif [20], [21] similarly to C-proteins and many other transcription regulators. To date, the Pfam database [22] contains 68 protein sequences which consist of a domain with the HTH motif followed by the C5-DNA MTase domain [23]. However, no crystallographic or NMR data about their structures are available as yet. The region between the RD and the MTase domain (residues 56–71) shares no similarity with any available high resolution model and contains 4 proline residues, suggesting this fragment to be potentially non-structured. The linker responsible for the interaction between the two domains of M.SsoII could play a crucial role in the functioning of M.SsoII in the cell. The N-terminal region (residues 1–71, *i.e.* the RD with the linker) determines the ability of M.SsoII to regulate transcription in the SsoII R–M system [12].

Since the mechanism of DNA methylation itself does not imply a dimer formation [24], most of DNA MTases exist in solution as monomers. On the contrary, transcription factors typically function as dimers and tetramers. Establishing the oligomeric state of M.SsoII in solution is therefore an important task with a clear functional implication. A deletion mutant representing only the MTase domain of M.SsoII has been found catalytically active but impossible to purify [25]. Because of this, another C5-DNA MTase, NlaX (M.NlaX), is used here as a control representing only the MTase domain. This enzyme shares 67% identity with the MTase domain of M.SsoII and has the same methylation specificity [11]. Due to the lack of additional domains, M.NlaX is transcriptionally inactive and can be regarded as a natural Δ(1–71) deletion mutant of M.SsoII.

In the present study, small-angle X-ray scattering (SAXS) is employed to determine the low resolution structures of apo-M.NlaX, apo-M.SsoII, and M.SsoII complexed with a 15-bp DNA duplex containing the methylation site (15met). The obtained models of M.SsoII and M.NlaX are compared with the structure of M.HhaI, a one-domain C5-DNA MTase, which has been studied extensively by X-ray crystallography [26], [27], [28]. The SAXS data along with the results of size exclusion chromatography (SEC) and dynamic light scattering (DLS) unambiguously point to the monomeric state of apo-M.SsoII and of its complex with 15met even at higher solute concentrations. The low resolution model of full-length M.SsoII reveals an extended but folded structure of the N-terminal region as a distinct domain tethered by a highly flexible linker to the MTase domain. A possible role of the linker flexibility for transcription regulation in the SsoII R–M system is discussed.

## Materials and Methods

### Protein expression and purification

*E. coli* strain M15 [pREP4] containing the plasmid pQMSsoII or pQMNlaX was grown at 37°C in LB medium with 30 µg/ml kanamycin and 50 µg/ml ampicillin to an A_600_ value of 0.6. Protein expression was induced with 0.7 mM isopropyl 1-thio-β-*D*-galactopyranoside, and the cell culture was kept for 20 h at 20°C. The cells were harvested by centrifugation. The cell pellets were resuspended in buffer A (50 mM Na-phosphate, 100 mM NaCl, 5 mM β-mercaptoethanol, 5% (w/v) glycerol, pH 7.0) and lysed by sonication. The lysate was clarified by centrifugation at 18,000 *g* and loaded onto **a** Heparin HP column (GE Healthcare) pre-equilibrated with buffer A. The target protein was eluted with a gradient from 0.1 to 1.0 M NaCl. In case of M.SsoII, the fractions containing this protein were loaded onto a HisTrap HP column (GE Healthcare) and eluted with a 20–400 mM imidazole gradient. The target proteins were concentrated and their purity was estimated using 12.5% SDS-PAGE. Because of cytotoxicity of M.SsoII, the yield of purified M.SsoII was 0.14 mg from 1 l of cell culture, two orders of magnitude lower than that of M.NlaX.

### DNA–protein complex formation

DNA duplex 15met containing the M.SsoII methylation site was formed by annealing an equimolar mixture of 5′-AGAGCCAGGAACCGA-3′ and 5′-TCGGTTCCTGGCTCT-3′ oligonucleotides (Metabion) in water, *i.e.* heating up to 70°C and cooling down slowly to room temperature. Complex formation between M.SsoII and 15met was carried out in buffer B (18 mM Tris-HCl, 136 mM NaCl, 3.5 mM β-mercaptoethanol, 10% (w/v) glycerol, pH 8.0) in the presence of AdoHcy. M.SsoII, AdoHcy, and the DNA duplex were mixed in ratio 1∶2∶1. The mixture was analysed by electrophoresis in 7% non-denaturing polyacrylamide gel. The gel was stained first with ethidium bromide (EtBr) to visualize DNA-containing bands and then with Coomassie Brilliant Blue to visualize protein-containing bands.

### Size exclusion chromatography

Size exclusion chromatography was performed on a Superdex 75 10/300 column (GE Healthcare). The column was pre-equilibrated and eluted with buffer C (50 mM Tris-HCl, 100 mM NaCl, 5 mM β-mercaptoethanol, 5% (w/v) glycerol, pH 8.0). M.SsoII was loaded in concentration 3.2 mg/ml in buffer D (20 mM Tris-HCl, 100 mM NaCl, 5 mM β-mercaptoethanol, 20% (w/v) glycerol, pH 8.0). M.NlaX was loaded in concentration 7.7 mg/ml in buffer E (50 mM Tris-HCl, 100 mM NaCl, 5 mM β-mercaptoethanol, 20% (w/v) glycerol, pH 8.0). The column was previously calibrated with ribonuclease A (13700 Da), carbonic anhydrase (29000 Da), ovalbumin (monomer of 43000 Da and dimer of 86000 Da), and bovine serum albumin (BSA, monomer of 66000 Da) in buffer F (50 mM Tris-HCl, 200 mM NaCl, 5 mM β-mercaptoethanol, 5% (w/v) glycerol, pH 8.0). A NaCl concentration of 200 mM in buffer F served to estimate the total volume of the column by measuring conductivity. Blue dextran in buffer F was used to estimate the column void volume.

### Dynamic light scattering

Dynamic light scattering measurements were performed using a ZetaSizer Nano-S (Malvern) with the laser wavelength of 633 nm in a quartz cuvette of 45 µl at 8°C. Samples of M.SsoII and M.NlaX were analyzed in 50 mM Na-phosphate buffer (pH 7.0) containing 5 mM β-mercaptoethanol with variations in glycerol or salt concentrations. The protein concentration was 0.5 mg/ml. The solution viscosities computed on the basis of glycerol concentration were 1.5217 cP and 1.8563 cP for water solutions containing 5% (w/v) and 15% (w/v) glycerol respectively. The range of concentrations appropriate to automatic choice of attenuation index was found to be 0.5–1.0 mg/ml for both proteins. The hydrodynamic diameter (*D*_h_) was evaluated by the Stokes–Einstein equation from the autocorrelation function of the DLS measurements following standard procedures and the average MM was estimated using default Mark–Houwink parameters for a hard sphere.

### SAXS measurements and data processing

Synchrotron radiation X-ray scattering data were collected on the EMBL X33 beamline at the DORIS III storage ring (DESY, Hamburg) [29]. Solutions of M.NlaX, M.SsoII, and M.SsoII–15met complex were measured for solute concentrations of 1.6–7.0 mg/ml, 1.0–3.3 mg/ml, and 1.0–1.5 mg/ml, respectively. A MAR345 image plate detector was used at the sample–detector distance 2.7 m and wavelength λ = 0.15 nm, covering the momentum transfer range 0.12<*s*<4.9 nm^−1^ (*s* = 4*π* sin*θ*/*λ*, where 2*θ* is the scattering angle). No radiation damage effects were detected by comparison of two data sets with 2-min exposure time. The data were averaged after normalization to the intensity of the incident beam, the scattering of the buffer was subtracted and the difference data were extrapolated to zero solute concentration using PRIMUS [30].

The radius of gyration *R*_g_ of solute protein molecule and the forward scattering *I*(0) were evaluated using the Guinier approximation at small angles (*s*<1.3/*R*_g_) [31] assuming the intensity was represented as *I*(*s*) = *I*(0) *exp*(−(*sR*_g_)^2^/3) and from the entire scattering pattern by the program GNOM [32]. In the latter case, the distance distribution functions *p*(*r*) and the maximum particle dimensions *D*_max_ were also computed. The molecular mass (MM) of the solute was evaluated by comparison of the calculated *I*(0) value with that of the standard solution of bovine serum albumin (MM of 66 kDa). The excluded volume of the hydrated protein molecule (*V*_p_) was calculated using the Porod approximation:
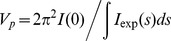
(1)in which the intensity *I*(*s*) was modified by subtraction of an appropriate constant from each data point to force the *s* ^−4^ decay of the intensity at higher angles following Porod's law [33] for homogeneous particles.

Low resolution *ab initio* models of M.NlaX and M.SsoII were generated by DAMMIN [34], representing the protein by an assembly of densely packed beads. Simulated annealing was employed to build a compact interconnected configuration of beads inside a sphere with the diameter *D*_max_ that fits the experimental data *I*_exp_(*s*) to minimize the discrepancy:
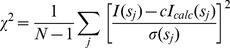
(2)where *N* is the number of experimental points, *c* is a scaling factor, *I*_calc_(*s_j_*) and σ(*s_j_*) are the calculated intensity and the experimental error at the momentum transfer *s_j_*, respectively. The common structural features of a model were determined by averaging the configurations from ten separate runs using the program DAMAVER [35].

An alternative model of M.SsoII was constructed by homology modeling. The MTase domain of M.SsoII was represented by the crystallographic model of M.HhaI, a homologous one-domain MTase (PDB entry: 2HMY) [28], referred further as “M.HhaI model” (see Table 1). The scattering from M.HhaI was calculated using the program CRYSOL [36] and was compared with the experimental data for M.NlaX, which also consists of only one MTase domain. To construct the model of full-length M.SsoII, the N-terminal fragment was added to the M.HhaI structure by two alternative ways using BUNCH [37]. In the first case, referred further as “hybrid M.HhaI model”, the entire fragment (71 residues) was represented by an interconnected chain of dummy residues (DRs) [38]. In the second case (“hybrid M.HhaI-R434 model”), a crystallographic model of a protein homologous to the RD, phage 434 repressor (PDB entry: 1PER) [39] was used to represent the first 55 residues of the N-terminal fragment as a rigid body. The homology model was attached to the MTase domain through a DRs linker, and its position and orientation relative to the MTase domain were refined. A simulated annealing protocol implemented in BUNCH was employed to generate clash-free native-like configurations either of the entire N-terminal fragment or, for the second case, of the linker, fitting the experimental scattering from M.SsoII.

**Table 1:** Models of M.NlaX, M.SsoII, and M.SsoII–15met complex constructed in the present work.

Object	Method of model construction	Model title
M.NlaX	*ab initio* modeling (DAMMIN)	*ab initio* model of M.NlaX
M.NlaX	homology modeling: the crystallographic structure of M.HhaI (PDB entry: 2HMY) as a template, CRYSOL for scattering calculation	M.HhaI model
M.SsoII	*ab initio* modeling (DAMMIN)	*ab initio* model of M.SsoII
M.SsoII	homology modeling: the crystallographic structure of M.HhaI (PDB entry: 2HMY)+the N-terminal region made of dummy residues	hybrid M.HhaI model
M.SsoII	homology modeling: the crystallographic structure of M.HhaI (PDB entry: 2HMY)+the crystallographic structure of phage 434 repressor (PDB entry: 1PER)+dummy residues linker in order to connect the two domains	hybrid M.HhaI-R434 model
M.SsoII–15met	*ab initio* modeling (MONSA)	*ab initio* model of M.SsoII–DNA complex
M.SsoII–15met	homology modeling: the crystallographic structure of M.HhaI–DNA complex (PDB entry: 3MHT)+the N-terminal region made of dummy residues	hybrid M.HhaI–DNA model
M.SsoII–15met	homology modeling: the crystallographic structure of M.HhaI–DNA complex (PDB entry: 3MHT)+the crystallographic structure of phage 434 repressor (PDB entry: 1PER)+dummy residues linker in order to connect the two domains	hybrid M.HhaI-R434–DNA model

The model of M.SsoII complex with a 15-bp DNA containing the methylation site (15met) was also constructed by two different methods, *ab initio* and homology modeling. In the first case, a multiphase bead modeling was performed *ab initio* using MONSA [40] which, similarly to DAMMIN, performs a search inside a spherical volume with the diameter *D*_max_. Simulated annealing was employed to find which bead belongs to which part of the complex (protein, DNA, or solvent) by simultaneous fitting of three scattering curves (two experimental curves, from M.SsoII alone and from the M.SsoII–15met complex, and a theoretical curve from the DNA duplex). The latter curve was computed by CRYSOL from the crystal structure of the 12-bp DNA duplex (crystallized in complex with M.HhaI, PDB entry: 3MHT) [27]. In the second case, the M.SsoII–15met complex structure was reconstructed by homology modeling using the crystallographic data for the M.HhaI complex with the 15-bp DNA duplex containing the methylation site (PDB entry: 3MHT). The missing N-terminal residues were added by two alternative ways as described above (see also Table 1), yielding “hybrid M.HhaI–DNA model” and “hybrid M.HhaI-R434–DNA model”. Both reconstructions using MONSA and BUNCH were performed assuming a 1∶1 stoichiometry of the M.SsoII–15met complex.

The flexibility of the N-terminal fragment of M.SsoII in apo-form and in the complex with 15met was assessed by the ensemble optimization method (EOM) [41], which allows for coexistence of different protein conformations contributing to the experimental scattering pattern. These conformers were selected using a genetic algorithm from a pool containing a large number of randomly generated models covering the protein configurational space. An ensemble pool of 10^5^ structures was generated by random additions of the N-terminal fragment (either a DR chain or the phage 434 repressor structure with the linker of ten DRs) to the “M.HhaI model”. The genetic algorithm was employed to find the subsets of these conformers, whose mixture fitted the experimental data. Multiple runs of EOM were performed and the obtained subsets were analyzed to yield the *R*_g_ distributions in the selected ensembles. In the case of the M.SsoII-15met complex the DNA duplex was added and kept in its crystallographic position relative to MTase domain of M.SsoII and the pool generation and EOM selection procedures were performed as described above for the M.SsoII alone.

## Results

### Association state and overall parameters of M.SsoII and M.NlaX apo-forms in solution

The association states of M.NlaX and M.SsoII have first been studied by SEC (Figure S1) and DLS. The apparent MMs of the proteins have been estimated using the column calibration against the standard proteins set. The calculated values correspond to apparent MM of 34 kDa and 41 kDa for M.NlaX and M.SsoII, respectively.

In the DLS experiments, slight increase of PDI up ∼0.24 in case of M.NlaX could be prompted by a higher viscosity of the more concentrated M.NlaX solutions. The size distribution of M.SsoII contained a single narrow peak with polydispersity index (PDI) below 0.1. Estimated *D*_h_ values varied in range 5.2–5.9 nm and 6.3–6.8 nm that corresponded to MMs of 32–43 kDa and 50–59 kDa for M.NlaX and M.SsoII, respectively. Note that the DLS calculations of MM are shape-dependent and this may lead to a somewhat overestimated MM of M.SsoII, which, as it will be seen later, is a rather elongated particle.

**Figure 1:**
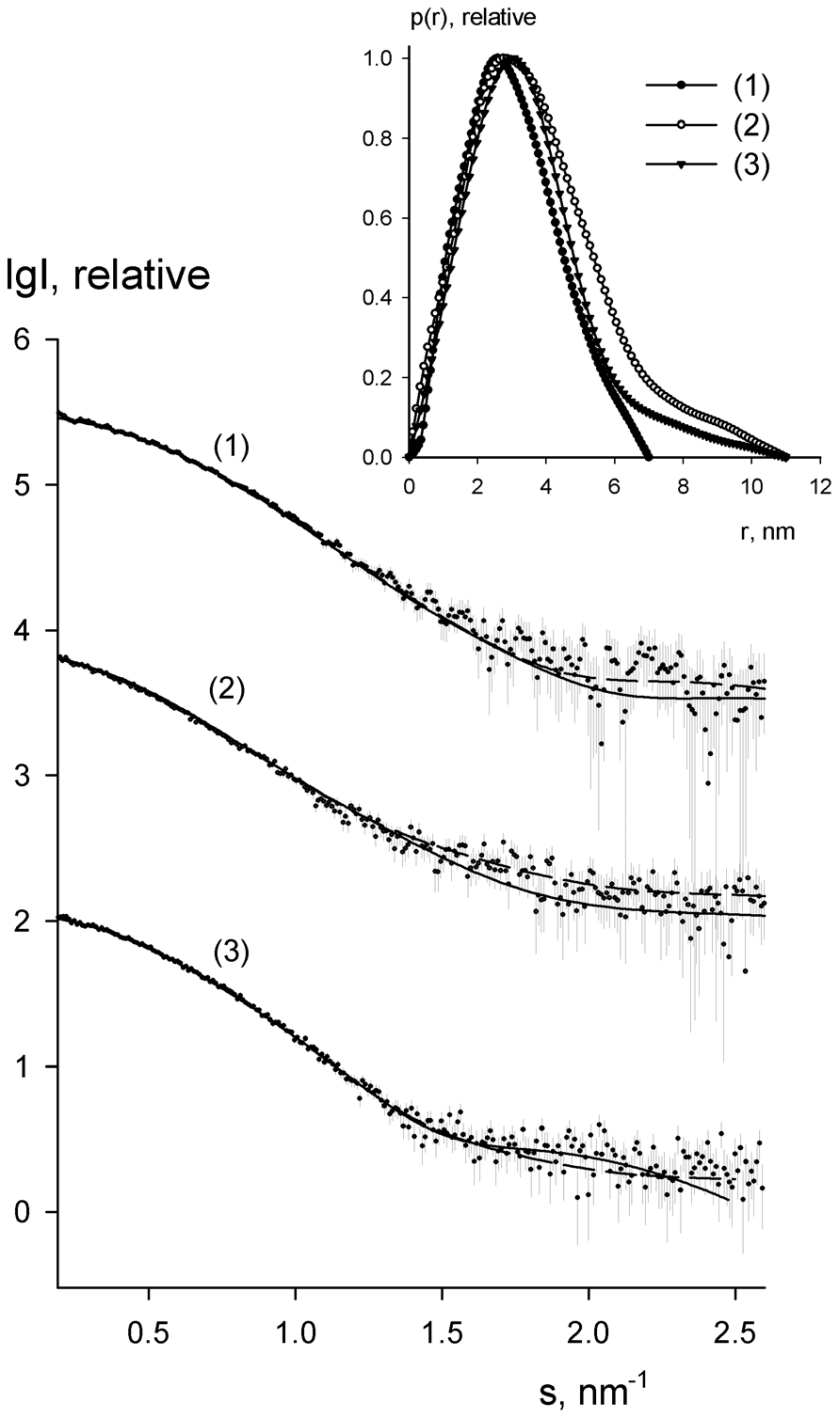
The experimental X-ray scattering data and the obtained fits. Curves 1–3 correspond to M.NlaX, M.SsoII and M.SsoII–15met complex,respectively. Experimental data are displayed as dots with error bars, the scattering from the typical *ab initio* models computed by DAMMIN or MONSA as full lines, and the calculated fits by CRYSOL (M.NlaX) or EOM (M.SsoII and the complex) as dashed lines. The plots display the logarithm of the scattering intensity as a function of momentum transfer. The distance distribution functions are presented in the insert.

The information about MM has also been obtained from the SAXS experiments. The X-ray scattering intensity patterns *I*(*s*) display no systematic changes with the solute concentration demonstrating no change in association state of M.NlaX and M.SsoII with concentration. The Guinier plots (initial portions of the scattering data in the coordinates ln *I* versus *s^2^*) are linear suggesting homogeneity of the samples. The processed scattering data and the computed distance distribution functions are displayed in Figure 1. The overall parameters extracted from the SAXS data are summarized in Table 2.

**Table 2:** Overall parameters calculated from SAXS^a^.

Sample	*c*, mg/ml	*R*_g_, nm	*D*_max_, nm	*V*_p_, nm^3^	MM_exp_, kDa	χ*_ab_*	χ*_rb_*	χ*_eom_*
**M.NlaX**	1.6–7.0	2.36±0.04	7.0±0.5	68±7	33±4	1.12	1.21^b^	–
**M.SsoII**	1.0–3.3	3.01±0.04	11.0±0.5	77±8	38±5	1.25	1.46	1.03
**M.SsoII–DNA**	1.0–1.5	2.79±0.04	11.0±0.5	85±10	45±6	1.10	1.78	1.05

(<emph type="italic">a</emph>) Notations: *R*_g_, radius of gyration; *D*_max_, maximum size of the particle; *V*_p_, excluded volume of the hydrated particle; MM_exp_, experimental molecular mass of the solute; χ_ab_, χ_rb_ and χ_eom_, values for the fit from *ab initio* models, from rigid body modeling using BUNCH and from EOM, respectively.

(<emph type="italic">b</emph>) in case of M.NlaX, χ_rb_ corresponds to the fit from the crystallographic structure of M.HhaI.

The experimental MM of M.NlaX (33±4 kDa) suggests that the protein is monomeric in solution (theoretical MM of the monomer 36.3 kDa). This is further corroborated by the excluded volume *V*_p_ of the particle 68±7 nm^3^, in agreement with an empirical finding for globular proteins that the hydrated volume in nm^3^ should numerically be about twice the MM in kDa. The experimental *R*_g_ and *D*_max_ (2.36±0.04 nm and 7.0±0.5 nm, respectively) point to a rather compact structure. The bell-shaped distance distribution function *p*(*r*) for M.NlaX (Figure 1, insert) is also consistent with a compact shape of the protein.

The experimental MM of M.SsoII (38±5 kDa) and its *V*_p_ (77±8 nm^3^) indicate that this protein is also monomeric in solution (theoretical MM of the monomer 44.9 kDa). In contrast to M.NlaX, *R*_g_ and *D*_max_ values (3.01±0.04 nm and 11.0±0.5 nm, respectively) point to an elongated shape of M.SsoII and the *p*(*r*) function for M.SsoII (Figure 1, insert) displays an asymmetric tail, typical for elongated particles.

### Stoichiometry and overall parameters of the M.SsoII complex with the 15-bp DNA containing the methylation site

The DNA construct with a length of 15 bp has been chosen since M.SsoII methylation site must be flanked with at least 4 bps from each side for effective methylation [11]. M.SsoII has been mixed with 15met in the presence of AdoHcy, as AdoMet or AdoHcy presence is necessary for the specific complex formation between M.SsoII and its methylation site [42]. The resulting mixture has been analyzed by native gel electrophoresis. Coomassie staining indicates complex formation without an excess of unbound protein while EtBr staining demonstrates a minor band corresponding to a very small amount of unbound DNA (Figure S2). The SAXS analysis (Figure 1 and Table 2) yield the experimental MM (45±6 kDa) and *V*_p_ (85±10 nm^3^) pointing to a 1∶1 stoichiometry for the M.SsoII–15met complex. Comparing the values of *R*_g_ (2.79±0.04 nm) and *D*_max_ (11.0±0.5 nm) for the complex with those for apo-M.SsoII, one can see that *D*_max_ of the complex remains the same as that of M.SsoII, but the *R*_g_ decreases, suggesting either the positioning of DNA in the central part of the complex or compaction of the entire structure. Still, the overall parameters of the M.SsoII–15met complex indicate an elongated shape and the *p*(*r*) function of the complex displays an asymmetric tail (Figure 1, insert).

### Two approaches for the molecule shape reconstruction

**Figure 2:**
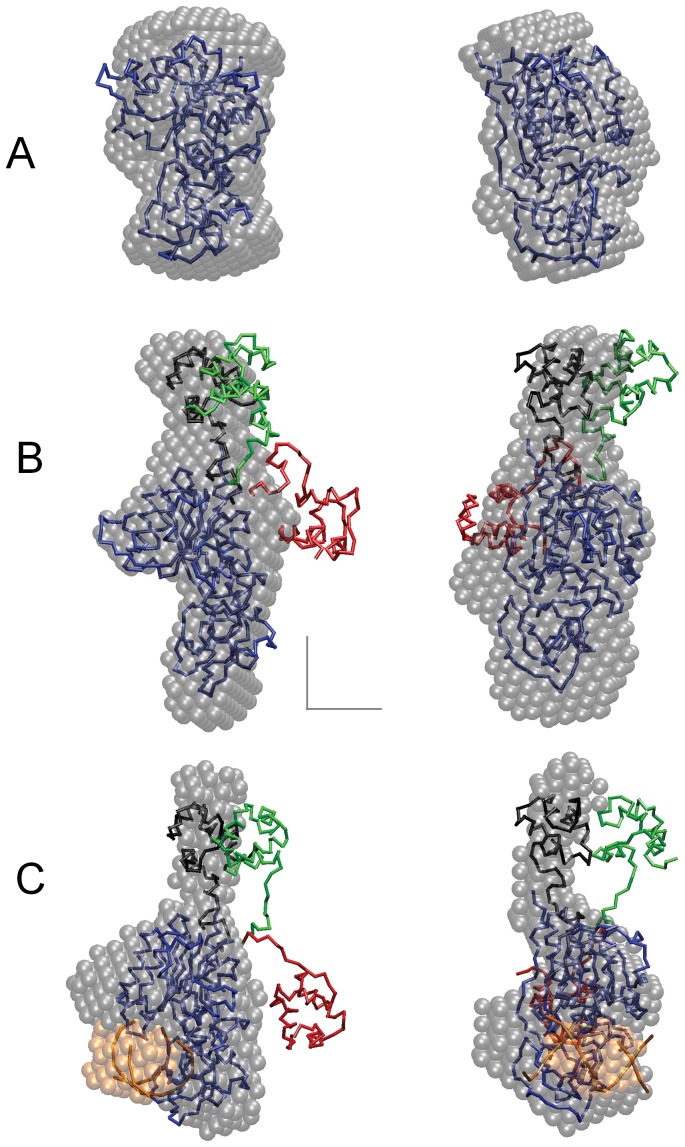
Structural models of M.NlaX, M.SsoII and its complex with DNA. (**A**) *Ab initio* bead model of M.NlaX obtained by DAMMIN (grey semitransparent spheres) superimposed with the crystallographic “M.HhaI model” (blue C_α_-traces). (**B** and **C**) *Ab initio* bead model of M.SsoII and M.SsoII–15met complex from DAMMIN/MONSA (gray semitransparent spheres correspond to M.SsoII, orange ones belong to the DNA) superimposed with the typical BUNCH model (black C_α_-traces) and with two typical conformations from an EOM ensemble (green and red C_α_-traces). The crystallographic “M.HhaI model” is displayed as blue C_α_-traces, the DNA molecule as orange helices. The right panels are rotated 90° counter-clockwise around the vertical axis. Scale bar, 2 nm.

The macromolecular shapes have been reconstructed by two different approaches: *ab initio* modeling (using only the experimental X-ray scattering data) and hybrid rigid body modeling (using a crystallographic model of a homologous protein as a template). *Ab initio* low resolution models of M.NlaX (Figure 2A) and M.SsoII (Figure 2B) have been generated by DAMMIN [34] (see Methods for details). For the homology modeling, the atomic model of C5-DNA MTase HhaI (M.HhaI) has been chosen for which the crystal structure is available. M.HhaI methylates the inner cytosine residue in the sequence 5′-GCGC-3′/3′-CGCG-5′, and the MTase domain of M.SsoII (as well as the whole M.NlaX) shares 41% identity with M.HhaI. The M.HhaI structure from PDB entry 2HMY [28] has been taken for modeling of M.NlaX and M.SsoII apo-forms. Both M.NlaX and M.HhaI consist of only the MTase domain. The full-length M.SsoII molecule contains in addition the N-terminal fragment, which was modeled (Figure 2B) in two alternative ways, as a DR-chain (“hybrid M.HhaI model”) or using a homologous structure [39], “hybrid M.HhaI-R434 model” (see model details in Methods).

The shape of the DNA–protein complex (Figure 2C) has also been independently reconstructed by *ab initio* and homology modeling. An *ab initio* model of the M.SsoII–15met complex has been generated by the program MONSA [40] (see Methods for details). For the homology modeling, the structure of M.HhaI in complex with the 15-bp DNA duplex containing its methylation site has been taken as a template (PDB entry: 3MHT) [27]. To reproduce full-length M.SsoII, the N-terminal fragment has been added to the M.HhaI structure by two alternative ways, similarly to the above construction of the apo-M.SsoII model (Table 1).

### *Ab initio* shapes of M.NlaX, M.SsoII, and M.SsoII complex with the 15-bp DNA containing the methylation site

A typical low resolution shape of M.NlaX reconstructed *ab initio* (Figure 2A) has the overall size of about 7 nm×4.4 nm×3 nm and fits the experimental data with discrepancy χ = 1.1 (Figure 1, curve 1, solid line). The scattering curve from the “M.HhaI model” calculated by CRYSOL (see Methods) agrees with the experimental data (χ = 1.2, Figure 1, curve 1, dashed line). This homology model overlaps well with the *ab initio* model (Figure 2A), suggesting that M.NlaX has the shape close to that of the “M.HhaI model”.

The *ab initio* low resolution shape of M.SsoII is displayed in Figure 2B and fits the experimental data with χ = 1.3 (Figure 1, curve 2, solid line). The model reveals two distinct domains, a “main” (larger) domain with the overall shape similar to that of M.NlaX, and an “additional” (smaller) domain presumably accounting for the N-terminal region of M.SsoII (missing in M.NlaX).

The *ab initio* two-component low resolution model of the M.SsoII–15met complex (Figure 2C) fits simultaneously the scattering patterns from M.SsoII and the M.SsoII–15met complex with the overall discrepancy χ = 1.1 (Figure 1, curve 3, solid line). This model demonstrates that the DNA duplex binds to the larger domain of M.SsoII, whereas the smaller domain corresponding to the N-terminal region of M.SsoII protrudes away from the DNA binding site.

### Rigid-body modeling of M.SsoII and M.SsoII complex with the 15-bp DNA containing the methylation site

To construct a more detailed model of M.SsoII, the “M.HhaI model” was treated as a rigid body, and the 71 N-terminal residues were represented as a chain of DRs yielding the “hybrid M.HhaI model”. Multiple runs of BUNCH (see Methods) starting from random initial configurations yield variable conformations of the N-terminal region, all providing good fits to the data with χ about 1.5. Some of the obtained models overlapped well with the *ab initio* model of M.SsoII (Figure 2B, black model), whereas the others displayed a tilted orientation of the N-terminal region with respect to the long axis of M.SsoII.

A similar approach was used to construct the model of the M.SsoII–15met complex. The M.HhaI complex with DNA has been taken as a rigid body and the missing N-terminal fragment of M.SsoII has been added by BUNCH, resulting in the “hybrid M.HhaI–DNA model”. Several BUNCH runs yielded an ensemble of solutions fitting the scattering data with χ about 1.8. Whereas the MTase domain of M.SsoII overlaps well with the larger domain of the *ab initio* model (Figure 2C), the presence of a variety of M.SsoII N-terminal region configurations suggests a significant flexibility of this region not only in the apo-M.SsoII but also in the M.SsoII–15met complex.

### Flexibility of the N-terminal region in apo-M.SsoII and in the M.SsoII complex with the 15-bp DNA containing the methylation site

The presence of disordered portions in apo-M.SsoII and in the M.SsoII complex with the 15 bp DNA is qualitatively supported by the Kratky plots (Figure S4). These plots display broad bell-shaped peaks, with the scattering intensities multiplied by s^2^ revealing upward trends at higher angles compared to the more downward trend observed for M.NlaX (where the N-terminus is missing). The increase of the higher angle portions of a Kratky plot is an indication of flexible portions in the particle.

**Figure 3:**
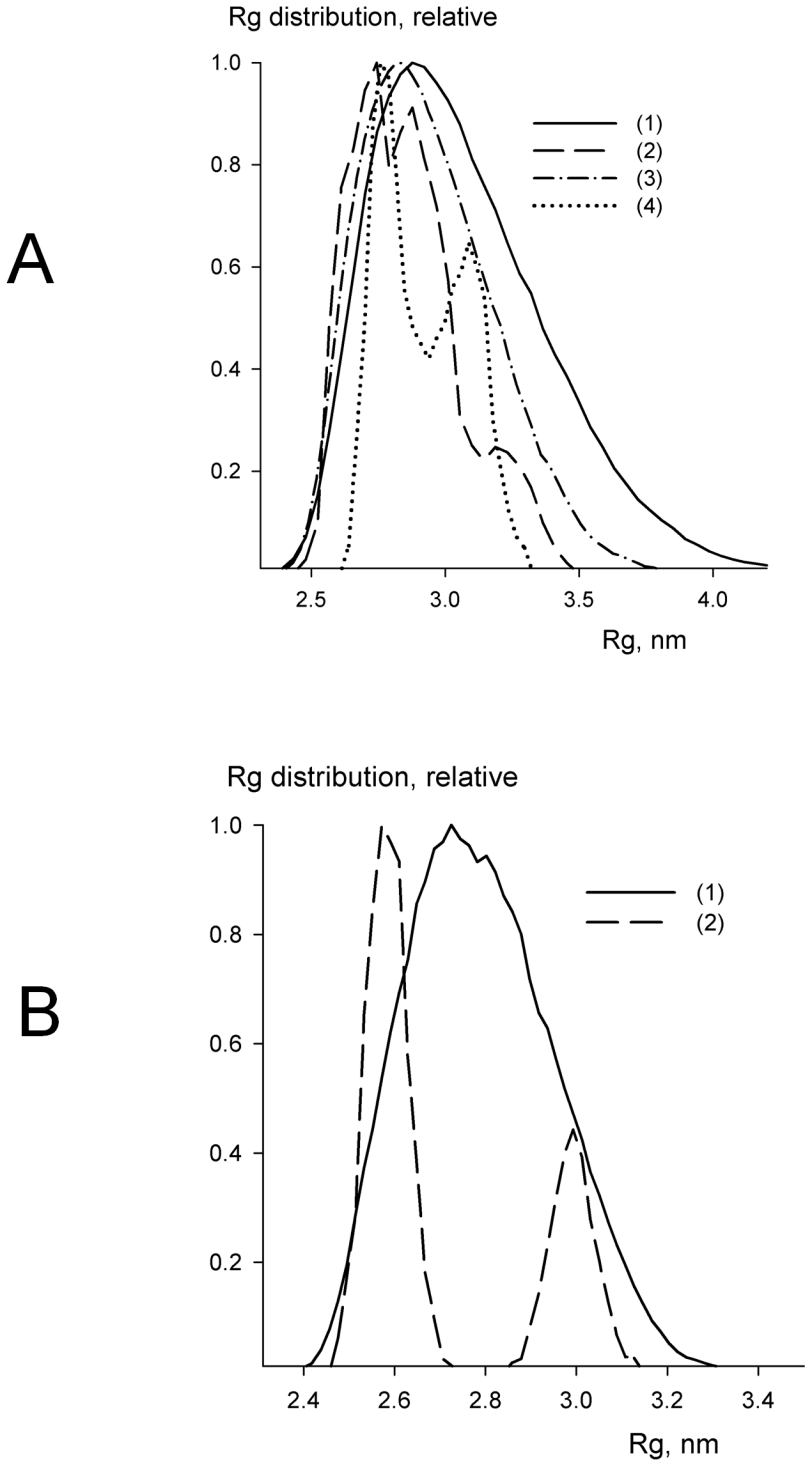
*R*_g_ distributions from EOM for M.SsoII. Solid and dashed-dot lines (curves 1 and 3) correspond to initial random pool; dashed and dotted lines (curves 2 and 4) to the selected ensembles (average of 50 independent EOM runs). Curves 1–2 correspond to the randomly generated N-terminal region of M.SsoII (“hybrid M.HhaI model”) and curves 3–4 were obtained with the “hybrid M.HhaI-P434 model”. The large width of the selected *R*_g_ distributions supports the flexibility of the N-terminal region in the M.SsoII molecule (**A**). *R*_g_ distributions from EOM for the M.SsoII–15met complex. The notations are the same as in (A) for the curves (1–2) (**B**).

The flexibility of the N-terminal region was quantitatively analyzed using EOM allowing for coexistence of multiple configurations in solution (see Methods). A typical optimized ensemble of “hybrid M.HhaI model” selected by EOM fits the data with χ = 1.0 (Figure 1, curve 2, dashed line). The *R*_g_ distribution of this ensemble (Figure 3A, curve 2) is nearly as broad as the distribution of randomly generated models (Figure 3A, curve 1) indicating that the N-terminal region is rather flexible. Moreover, the *R*_g_ distribution derived from EOM displays a bimodal profile with the major fraction of relatively compact models (*R*_g_ about 3 nm) and a minor fraction of models with *R*_g_ about 3.2 nm, where the N-terminal region configuration is extended. A typical optimized ensemble of “hybrid M.HhaI–DNA model” selected by EOM (Figure 2B, green, red models and Figure S3) fits the data with χ = 1.05 (Figure 1, curve 3, dashed line). The *R*_g_ distribution of the “hybrid M.HhaI–DNA model” is also bimodal but the ratio between the two fractions is shifted towards the more compact conformation (Figure 3B, curve 2).

Finally, to test whether the N-terminal region presents a disordered chain or has a defined tertiary structure, the “hybrid M.HhaI-R434 model” was constructed, where the RD is represented as a rigid body by the homologous phage 434 repressor structure and only the linker which connects the RD to the “main” domain is flexible. The models created by BUNCH (see Methods) fit the data with χ = 1.79 and display varying orientations of the N-terminal region to the long axis of the M.SsoII “main” domain, similar to those obtained by using a completely flexible chain of the N-terminal residues. EOM calculations for the “hybrid M.HhaI-R434 model” yield a good fit (χ = 1.0) and provide a broad *R*_g_ distribution of the selected models (Figure 3A, curve 4). The variety of configurations of the N-terminal region residues is also compatible with the experimental data (Figure 2C, green, red models and Figure S3) and reflect the flexibility of this region taken as a single rigid body.

## Discussion

In the earlier association state studies of DNA MTases in solution, diverging results have been reported. Some of DNA MTases are shown to exist predominantly as dimers, namely M.RsrI [43], M.MspI [44], and the Q237W mutant of M.HhaI [45], while some others remain monomeric, for example M.BamHI [46] and M.EcoRI [47]. In the present work, the oligomerization behavior of apo-M.SsoII was examined by SEC, DLS, and SAXS in a concentration range of 0.5–3.2 mg/ml. The data from all these methods are fully consistent with the monomeric state of apo-M.SsoII, and the protein remains monomeric upon binding to the 15met duplex.

Similar to most transcription factors bound to promoters comprising an inverted repeat [48], [49], [50], M.SsoII has to control its specificity and activity either by DNA-mediated oligomerization or by dimer self-assembly prior to the interaction with the promoter. Unfortunately, the M.SsoII complex with the regulatory site yielded a non-homogeneous reaction mixture and therefore could not be studied by SAXS. However, it has been shown recently that M.SsoII binds to a long DNA duplex (60-bp) with the regulatory site forming a complex with a stoichiometry protein∶DNA = 2∶1 [51]. No direct contacts between the protein subunits in the complex were observed, and, given the monomeric state of apo-M.SsoII in solution, it seems unlikely that the protein assembles into dimers prior to the interaction with the regulatory site. It is therefore conceivable that DNA plays the major role in the formation of the M.SsoII complex with the regulatory site of the promoter region.

A typical C5-DNA MTase domain consists of 2 subdomains separated by a DNA-binding cleft. The larger subdomain comprises 10 motifs conservative for all C5-DNA MTases [52] and contains the AdoMet-binding site as well as the binding site for the target cytosine residue. The other subdomain (small, target recognition domain, TRD) carries a sequence, which is unique in every MTase and is responsible for the substrate specificity. Thus, the large subdomains of different C5-DNA MTases share high similarity in primary and tertiary structure while the small subdomains vary substantially in size and spatial structure [24].

The *ab initio* shapes of M.NlaX (Figure 2A) and apo-M.SsoII (Figure 2B) obtained from SAXS differ substantially from each other. The more elongated shape of M.SsoII demonstrates itself already in the noticeable increase of *R*_g_ and *D*_max_ (Table 2) and in the asymmetric tail at the higher *r* of its *p*(*r*) distribution (Figure 1, insert). The M.SsoII bead model displays two distinct domains, the “main” one and the “additional” one, and the latter is absent in the M.NlaX model. The low resolution structure of M.NlaX is consistent with the crystallographic model of M.HhaI [28], a one-domain homologue of M.NlaX and M.SsoII. This similarity was employed to construct hybrid models of M.SsoII, representing the N-terminal region either as a flexible chain of DRs or as a rigid homology model using connected to the “main” domain through a flexible linker.

The “main” domain of M.SsoII in apo-form as well as in the complex with 15met matches the overall shape of M.NlaX and encompasses well the C_α_-traces of M.HhaI conformations simulated for the template of the full-length M.SsoII. The prominent structural peculiarity of the “main” domain, its V-like cleft, is empty in the model of apo-M.SsoII and encloses the “DNA” beads in the M.SsoII–15met complex (Figure 2B–C). The mutual arrangement of “DNA” and “protein” beads is compatible with the MTase domain organization observed in the crystal structures of M.HhaI and M.HaeIII complexes with DNA [24], [26], [53], where the two protein subdomains embrace the DNA molecule. MTase binding to DNA containing its methylation site is known to mediate substantial conformational changes [26], [53] leading to a more compact protein structure. The smaller *R*_g_ value of the M.SsoII–15met complex model compared with the apo-M.SsoII strongly supports the identification of the “main” domain as a structural region corresponding to 72–379 residues of M.SsoII responsible for its methylation function.

The “additional” domain of the *ab initio* shape accommodates the N-terminal residues missing in the M.NlaX and M.HhaI sequences. The beads of this region are absent in the SAXS model of M.NlaX and the simulated conformations of M.HhaI also do not overlap with these beads. Sequence analysis of M.SsoII suggests that only a minor part of the N-terminal region is disordered while the major part (residues 1–55) represents a domain with a pronounced spatial structure. This assumption has been confirmed by circular dichroism spectroscopy combined with gel-shift assay [51]. A deletion mutant representing the N-terminal region of M.Ecl18kI (differs from M.SsoII only by a single residue, Ile56Met) demonstrates a pronounced secondary structure and also retains the ability to bind specifically to the regulatory site, although with a lower affinity.

**Figure 4:**
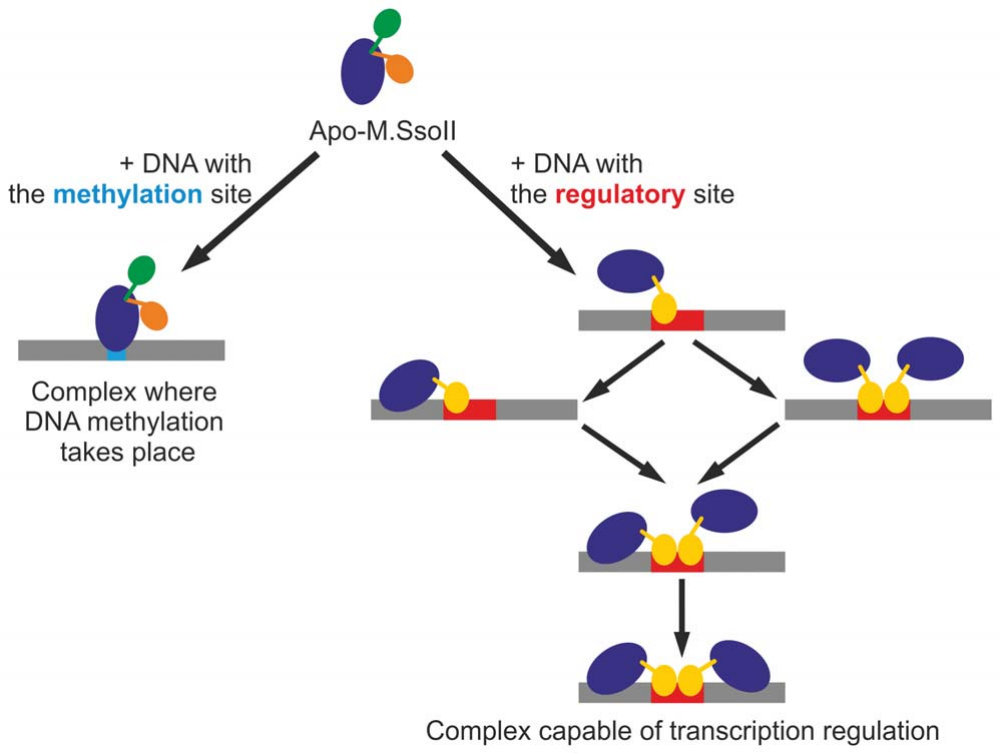
Possible role of the linker flexibility in M.SsoII binding to target DNA. DNA is in grey, the methylation site is in cyan, the regulatory site is in red. The MTase domain is shown in blue. Apo-M.SsoII demonstrates high linker flexibility which results in coexistence of different conformations of the N-terminal region (shown by green and orange). Upon M.SsoII binding to the methylation site, the dominant RD conformers keep the same orientations as in the apo-form. Binding to the regulatory site is supposed to be a multi-stage process which results in M.SsoII dimer formation where both domains of each M.SsoII subunit are bound to the same DNA duplex. Such structural organization is likely to fix both domains in a certain position in relation to each other. As we do not know whether the N-terminal region position in the latter complex is similar to any of the dominant RD conformers in the M.SsoII complex with the methylation site, the N-terminal region is shown by another (yellow) color in the latter case.

The models obtained with the “hybrid M.HhaI model” display a pronounced variability of the N-terminal fragment of M.SsoII (Figure 2B). The experimental scattering is also well described by rigid body movements of the “hybrid M.HhaI-R434 model”, suggesting that the latter model is an adequate representation of the M.SsoII N-terminal region. Thus, the full-length M.SsoII can be described as a C5-DNA MTase domain connected through a flexible linker to a folded RD which acts as a transcription regulator. This is represented schematically in Figure 4, where the “main” (MTase) domain of apo-M.SsoII is displayed in blue while the different possible orientations of the “additional” regulatory domain are depicted in green and orange.

The RD mobility is explored in more detail by the analysis of multiple coexistent conformers generated for the N-terminal residues using EOM. Comparison of the *R*_g_ distributions calculated for the apo-M.SsoII (Figure 3A) and for the M.SsoII–15met complex (Figure 3B) reveals further details of the linker flexibility. Both distributions are bimodal and the *R*_g_ values of both modes for the complex are smaller than those for the apo-form, in agreement with the observed decrease of the overall parameters of the M.SsoII–15met complex. Thus, the dominant RD conformers keep the same orientations in the complex as in the apo-form. Simultaneously, the two modes appear to be more distinct in the *R*_g_ distribution for the complex, suggesting a somewhat more restricted conformational space for RD in the complex.

High linker flexibility in M.SsoII has recently been suggested on the basis of protein–protein crosslinking experiments [54], and the present work provides a direct structural evidence by a completely different technique, SAXS. The linker flexibility is likely to play an important role for the ability of M.SsoII to regulate transcription. This ability is based on M.SsoII binding to the regulatory site in the promoter region of the SsoII R–M system [12]. M.SsoII forms a stable complex with the regulatory site, which competes with RNA polymerase and therefore prevents transcription of *ssoIIM* gene [14], [55]. This effect decreases the concentration of M.SsoII in the cell thus forming a regulatory circuit with a negative feedback. The *ssoIIR* gene promoter is weaker than the *ssoIIM* gene promoter and therefore repression of *ssoIIM* transcription stimulates *ssoIIR* gene transcription indirectly [14], [55]. Thus, the regulatory activity of M.SsoII is in anticorrelation with its main function, DNA methylation. Switching between these two functions should be provided by M.SsoII binding either to the methylation site or to the regulatory site (Figure 4).

M.SsoII complex formation with the regulatory site is expected to be a multi-stage process (Figure 4). An unusual structure of the complex has been proposed on the basis of footprinting and crosslinking experiments: the M.SsoII N-terminal regions are bound to the regulatory site while the MTase domains are bound to DNA flanking the regulatory site (Figure 4) [54]. We suppose that the first step of the complex formation should be the RD binding to the regulatory site followed then by the MTase domain binding to the same DNA duplex in a non-specific manner which provides higher stability to the complex. In general, a high level of non-specific binding is typical for M.SsoII [56]. Such a structure where both M.SsoII domains are bound to the same DNA duplex is possible only in the case where the linker between the domains is extremely flexible. Indeed, its flexibility is confirmed in the present work. Since the catalytic centre in the M.SsoII complex with the regulatory site is occupied by non-specific DNA, M.SsoII can not bind to the methylation site anymore. Thus, the linker flexibility is a key structural feature which provides formation of the stable complex capable of transcription regulation and therefore switches off the methylation function of M.SsoII.

## Supporting Information

Figure S1 **Determination of the MM by size exclusion chromatography (SEC).** (A) and (B) present the SEC data for (**A**) M.NlaX and (**B**) M.SsoII. (**C**) MM estimation using the calibration curve. *K*_av_ = (*V*_e_−*V*_0_)/(*V*_t_−*V*_0_), where *V*_e_ is elution volume of the sample, *V*_0_ is the column void volume, and *V*_t_ is the column total volume.
(TIF)

Figure S2 **Complex formation between M.SsoII and the 15-bp DNA containing the methylation site.** The native gel data correspond to 22 µM M.SsoII, 22 µM 15met and 44 µM AdoHcy (Coomassie staining, Lane 1; EtBr staining, Lane 2).
(TIF)

Figure S3 **EOM analysis of the M.SsoII data.** Typical selected ensembles for M.SsoII are presented in the left panel and M.SsoII–15met complex in the right panel. The MTase domain of M.SsoII is shown with magenta C_α_-traces, the restored N-terminal region with blue, green, red, grey, and cyan colors. The DNA molecule is displayed as yellow helices.
(TIF)

Figure S4 **Kratky plots corresponding to the data in** **Figure 1** **.** Experimental SAXS profiles were appropriately displaced along the logarithmic axis for better visualization.
(TIF)
