# Supplementary figures and images for: Flexibility of the Linker between the Domains of DNA Methyltransferase SsoII Revealed by Small-Angle X-Ray Scattering: Implications for Transcription Regulation in SsoII Restriction–Modification System
Source: PLoS One. 2014 Apr 7;9(4):e93453. doi: 10.1371/journal.pone.0093453 (PMC3978073; doi:10.1371/journal.pone.0093453)

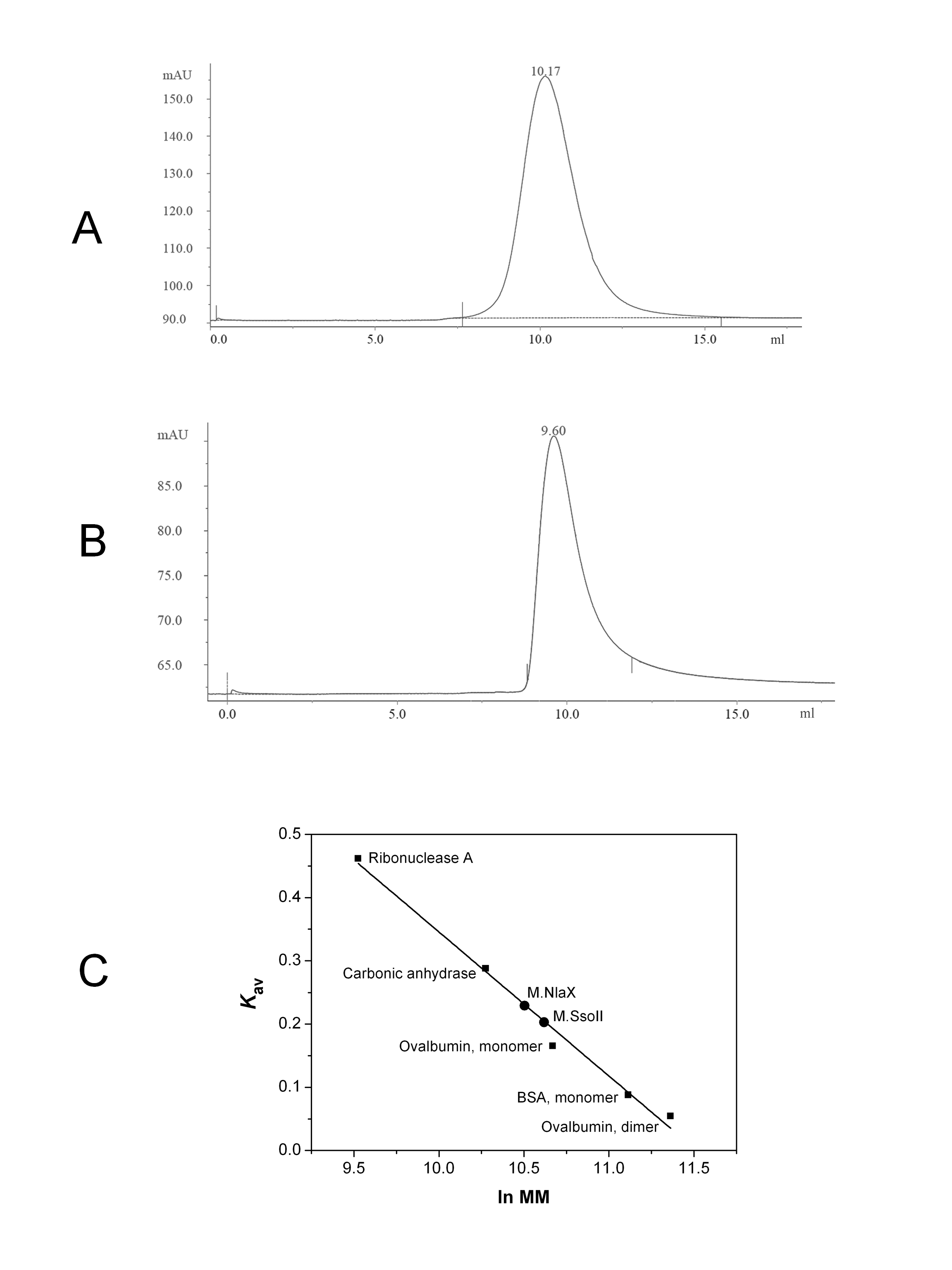

Supplement: Figure S1 — Determination of the MM by size exclusion chromatography (SEC). (A) and (B) present the SEC data for (A) M.NlaX and (B) M.SsoII. (C) MM estimation using the calibration curve. K av = (V e−V 0)/(V t−V 0), where V e is elution volume of the sample, V 0 is the column void volume, and V t is the column total volume. (TIF) [file pone.0093453.s001.tif]

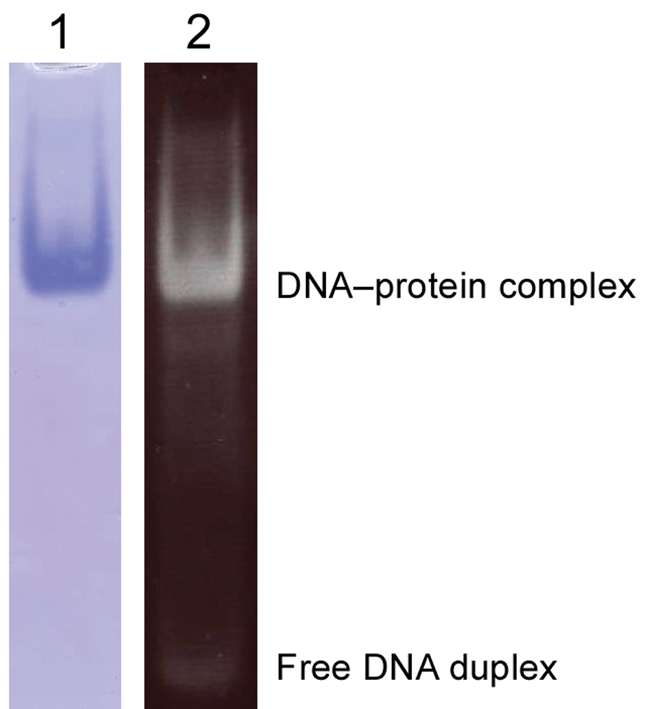

Supplement: Figure S2 — Complex formation between M.SsoII and the 15-bp DNA containing the methylation site. The native gel data correspond to 22 µM M.SsoII, 22 µM 15met and 44 µM AdoHcy (Coomassie staining, Lane 1; EtBr staining, Lane 2). (TIF) [file pone.0093453.s002.tif]

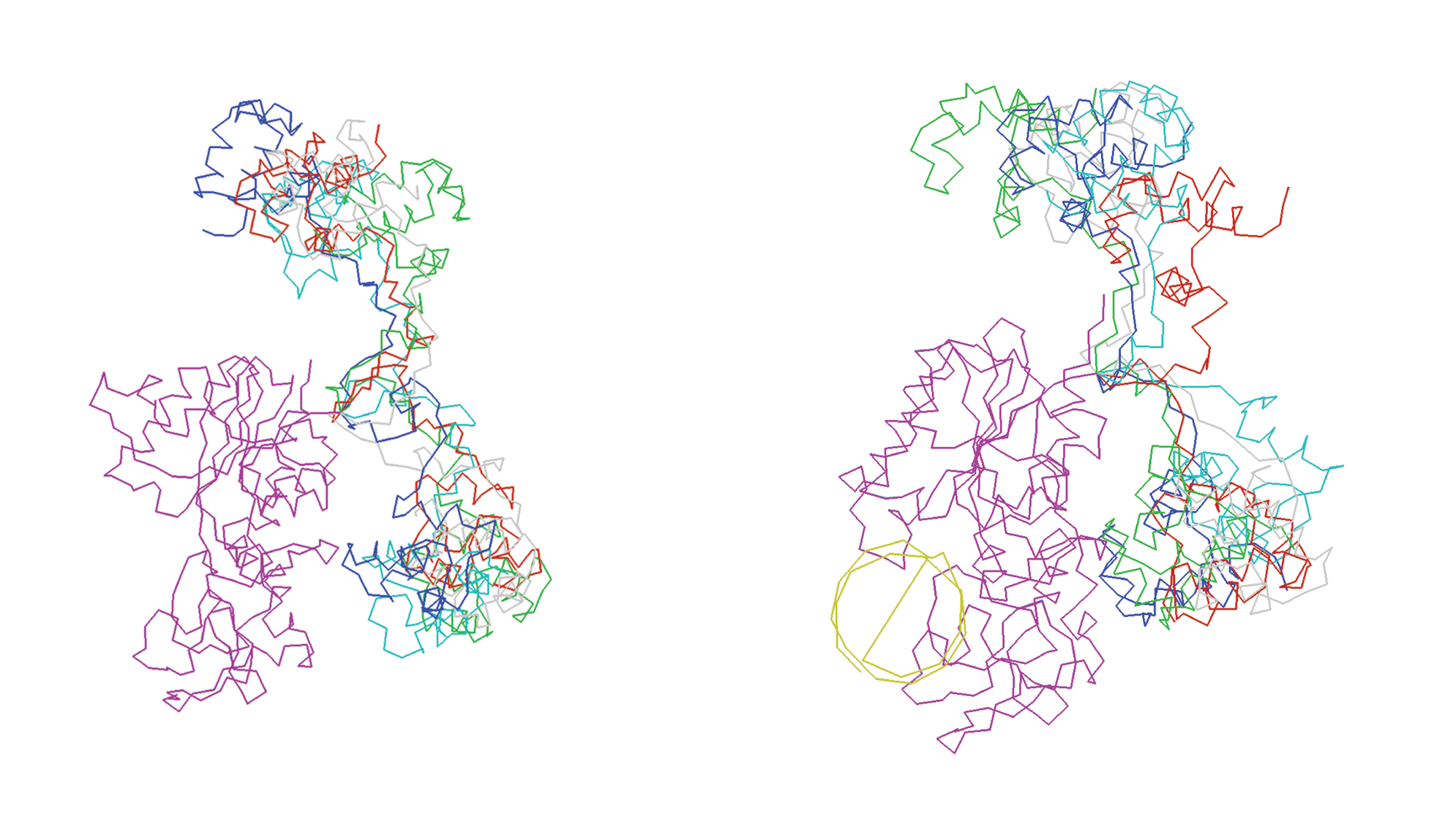

Supplement: Figure S3 — EOM analysis of the M.SsoII data. Typical selected ensembles for M.SsoII are presented in the left panel and M.SsoII–15met complex in the right panel. The MTase domain of M.SsoII is shown with magenta Cα-traces, the restored N-terminal region with blue, green, red, grey, and cyan colors. The DNA molecule is displayed as yellow helices. (TIF) [file pone.0093453.s003.tif]

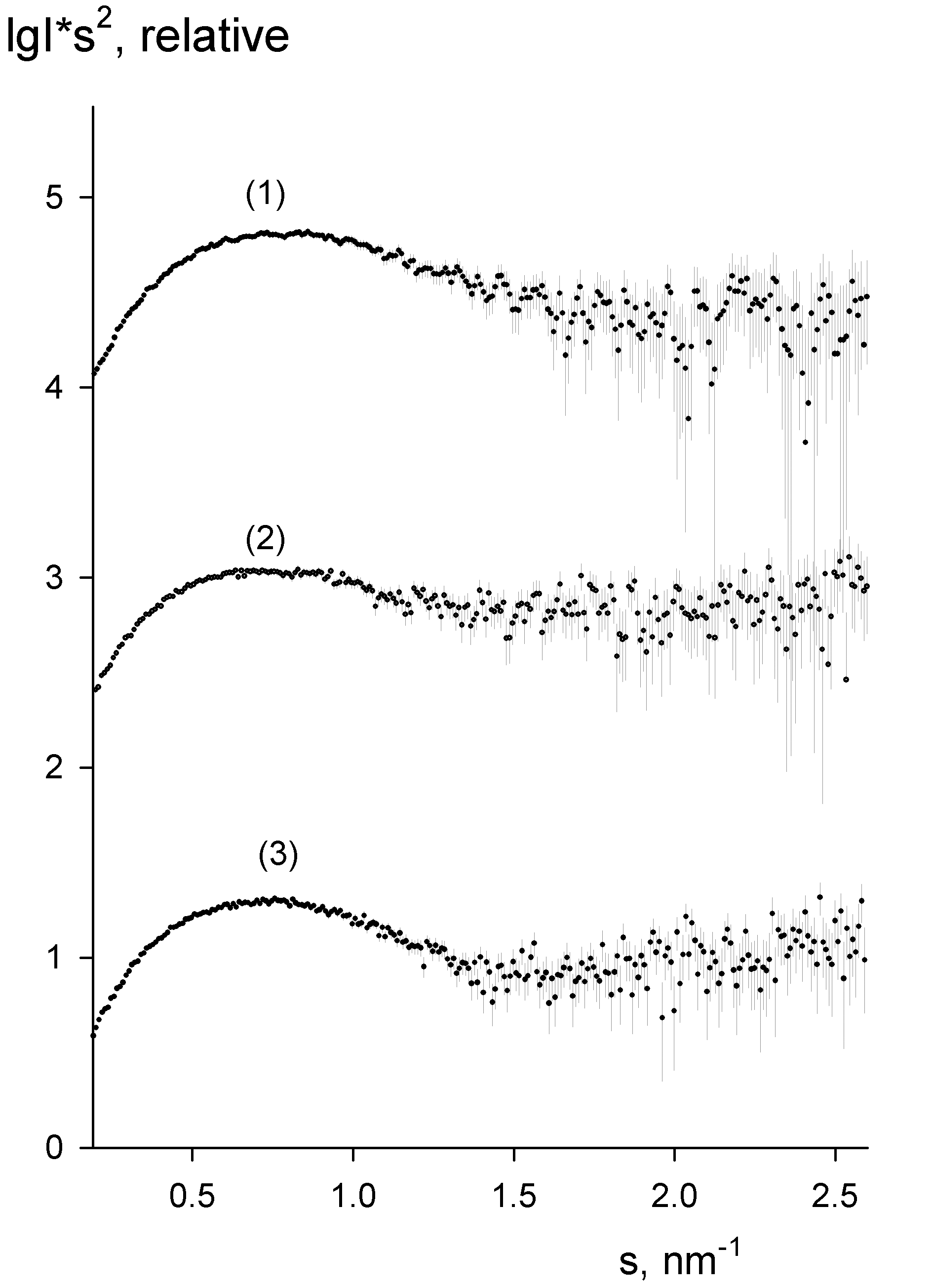

Supplement: Figure S4 — Kratky plots corresponding to the data in Figure 1 . Experimental SAXS profiles were appropriately displaced along the logarithmic axis for better visualization. (TIF) [file pone.0093453.s004.tif]
